# Microbiota and quality profiling of fermented goat meat sausages (sucuk) under nitrite-reduced and mixed-culture strategies

**DOI:** 10.1007/s11274-026-04804-5

**Published:** 2026-01-27

**Authors:** Damla Bilecen Şen, Pelin Ertürkmen, Duygu Alp Baltakesmez

**Affiliations:** 1 Department of Food Engineering, Faculty of Engineering Architecture, Burdur Mehmet Akif Ersoy University, Burdur, Turkey; 2 Department of Food Processing, Vocational School of Burdur Food Agriculture and Livestock, Burdur Mehmet Akif Ersoy University, Burdur, 15030 Turkey; 3 Department of Gastronomy and Culinary Arts, School of Tourism and Hospitality Management, Ardahan University, Ardahan, Turkey

**Keywords:** Microbiota, *Lactobacillaceae*, Sausage, Goat meat, Nitrite reduction, Bioprotective potential

## Abstract

The bioprotective activity of lactic acid bacteria (LAB) to modulate the microbiota and quality of nitrite-reduced fermented goat meat sucuk was investigated. Antagonistic activity of LAB strains against foodborne pathogens was evaluated using agar well diffusion, spot-on lawn, and cross-streak assays. Three LAB isolates affiliated with the genera *Weissella*, *Limosilactobacillus*, and *Lactiplantibacillus*, exhibiting inhibition zones > 18 mm, were selected and applied as a mixed culture (MC; 2:1:1). Sucuk formulations with 150, 75, and 0 ppm sodium nitrite were produced in the presence or absence of a MC and analyzed during fermentation (days 0 and 7) and refrigerated storage (days 7 and 14). Among the treatments, 75 ppm nitrite combined with MC (75-MC) exhibited the highest LAB counts, enhanced acidification (pH 4.7 on fermentation day 7), inhibited pathogens and spoilage microorganisms, and improved moisture and color stability (> 90% of initial L^*^ and a^*^), with a significant treatment × day interaction (*P* < 0.05). Metagenomic analysis of the 16 S rRNA (V3–V4) and ITS2 regions revealed a LAB-dominated sucuk microbiota, characterized by *Levilactobacillus* (69.5%), *Lactiplantibacillus* (12.1%), *Psychrobacter* (8.8%), and *Lacticaseibacillus* (3.0%) among bacteria, and *Yarrowia* (46%), *Kurtzmaniella* (11.8%), *Geotrichum* (6.7%), and *Cladosporium* (5.5%) among fungi. This microbial composition was associated with enhanced microbial stability and technological quality, while mixed-culture strategies under nitrite-reduced conditions promoted a *Lactobacillaceae*-enriched microbiota, highlighting their potential role in bioprotection and product quality.

## Introduction

Traditional dry-fermented sausages (sucuk) are typically produced through spontaneous fermentation under favorable environmental conditions such as ambient temperature, airflow, and relative humidity (Kaban et al. 2022). However, this traditional approach commonly causes variability in the final product due to differences in raw material composition and the unpredictable activity of indigenous microbiota (Sawant et al. 2025). To overcome these issues and improve process control, the use of defined starter cultures has become increasingly important (Yalçın and Ertürkmen 2024). Among lactic acid bacteria (LAB), members of the *Lactobacillaceae* family, particularly *Lactiplantibacillus plantarum* and *Latilactobacillus sakei*, are widely applied as starter cultures due to their metabolic flexibility and bioprotective effects (Pavli et al. 2020; Oliveira et al. 2021). Their effectiveness is greater when strains are isolated from traditional fermented foods or natural habitats and selected for well-defined physiological and technological attributes (Yalçın and Ertürkmen 2024). In addition, certain probiotic LAB strains inhibit foodborne pathogens and enhance sensory qualities (Leroy and De Vuyst 2016; García et al. 2020). Thus, they help preserve traditional flavor while contributing nutritional and protective benefits (Toldrá et al. 2021).

The antimicrobial effect of LAB is mainly associated with the production of organic acids, fatty acids, hydrogen peroxide, ethanol, and antifungal peptides, which often act synergistically (Sharma and Lee 2025). Among LAB, *Weissella cibaria* has received considerable attention due to its pronounced antimicrobial and antifungal properties (Alp and Kuleaşan 2020). This species, first described in 2002 (Kim et al. 2017; Dai et al. 2018), is commonly found in traditional fermented foods and the human gastrointestinal tract, where it produces exopolysaccharides associated with antibacterial, antifungal, anti-inflammatory, and anticancer effects (Kang et al. 2020; Lee et al. 2020; Du et al. 2023). In addition to *W. cibaria*, other LAB such as *Limosilactobacillus fermentum* have also been reported to inhibit a broad range of bacteria (Li et al. 2015) and fungi (Behbahani et al. 2023). Given the bioprotective potential of these LAB, in vitro antimicrobial assays including agar well diffusion, spot-on lawn, and cross-streak methods are widely used due to their simplicity and reproducibility (Hossain 2024).

In fermented sucuk, the moderately acidic pH (~ 4.6) provides conditions that support acid-tolerant yeasts such as *Candida* spp., *Debaryomyces hansenii*,* Saccharomyces cerevisiae*, and *Rhodotorula* spp. These yeasts pose a major spoilage risk, as they can proliferate under low water activity, acidic conditions, and refrigeration. The problem is made worse by high microbial loads in raw meat, inadequate fermentation and contamination after processing (Rani et al. 2023). Conventional control strategies, such as pH reduction, drying, nitrite addition, and anaerobic packaging are widely used; however, biological preservation using protective LAB has recently gained attention as a natural and effective alternative **(**Chauhan and Rao 2024).

The microbiota of fermented foods can be investigated using both culture-dependent and culture-independent methods. While culture-based approaches provide useful insights, they often overlook fastidious or uncultivable microorganisms (Nieminen et al. 2012; Buhutia et al. 2021). In contrast, metagenomic analysis enables comprehensive profiling of microbial populations, allowing the detection of rare taxa and supporting the optimization of fermentation and product quality (Wang et al. 2021; Franciosa et al. 2021; Soyuçok 2025). Recent metagenomic studies have revealed the complex microbial ecosystems of various fermented meat products, including traditional Turkish-style dry-fermented sausage (Soyuçok 2025), Italian dry-fermented sausages and Brazilian salami-type sausages (Franciosa et al. 2021; Degenhardt et al. 2021), and Mediterranean spontaneously fermented sausage products (Barbieri et al. 2021; Bassi et al. 2022).

Goat meat, with its low fat content and favorable fatty acid composition, is considered a healthier red meat option (Şengül and Bilecen Şen 2025). Its lean structure and reduced water-holding capacity may also limit fungal contamination. The development of nitrite-reduced fermented sucuk using goat meat, combined with well-characterized LAB starter cultures, therefore represents a promising strategy for producing safer, healthier and microbiologically stable products. However, limited information exists regarding the microbiota of nitrite-reduced fermented sucuk, a growing area of interest due to health-driven reformulation strategies.

This study investigated the microbiota dynamics of nitrite-reduced fermented goat meat sucuk by combining culture-dependent assay with metagenomic analyses (16 S rRNA V3–V4 and ITS2) and demonstrated the contribution of a mixed LAB culture (*Lpb. plantarum*, *Lb. fermentum*, *W. cibaria*) to improved fermentation performance, microbial safety, and clean-label reformulation strategies.

## Materials and methods

### Preparation and standardization of LAB strains and pathogens

Six LAB strains (*Lacticaseibacillus casei*,* Lactobacillus coryniformis*,* Lb. fermentum*,* Lpb. plantarum*,* Leuconostoc lactis*, and *W. cibaria*), previously isolated and characterized for probiotic properties including bacteriocin and organic acid production as well as inter-strain and pathogen interactions (Alp 2018; Alp and Kuleaşan 2020), were assessed for their antagonistic activity against selected foodborne pathogens. The bioprotective activity of LAB strains was tested against eight pathogens: *Escherichia coli* ATCC 25,922, *Staphylococcus aureus* ATCC 29,213 and ATCC 6538, *Salmonella* Typhimurium ATCC 14,028, *Listeria monocytogenes* ATCC 7644, *Enterococcus faecalis* ATCC 19,433, *Candida albicans* ATCC 14,053 and ATCC 10,231. Pathogen bacteria were cultivated in Tryptic Soy Broth (TSB; Merck, Germany) at 37˚C for 24 h, while yeast strains were cultivated in Yeast Peptone Dextrose Broth (YPD; Sigma-Aldrich, USA) at 30˚C for 72 h. LAB strains were activated in De Man Rogosa and Sharpe (MRS; Merck, Germany) Broth at 30˚C for 18 h. Fresh cultures were harvested, washed, and resuspended in sterile saline solution (0.85% NaCl), followed by centrifugation at 5000 rpm at 4˚C for 20 min. Cell suspensions were standardized to 0.5 McFarland turbidity, corresponding to approximately 1.5 × 10⁸ CFU/mL for bacteria and 1.5 × 10⁶ CFU/mL for yeasts (Souza et al. 2020).

### *In vitro* evaluation of bioprotective activity of LAB strains using different assays

**Fig. 1:**
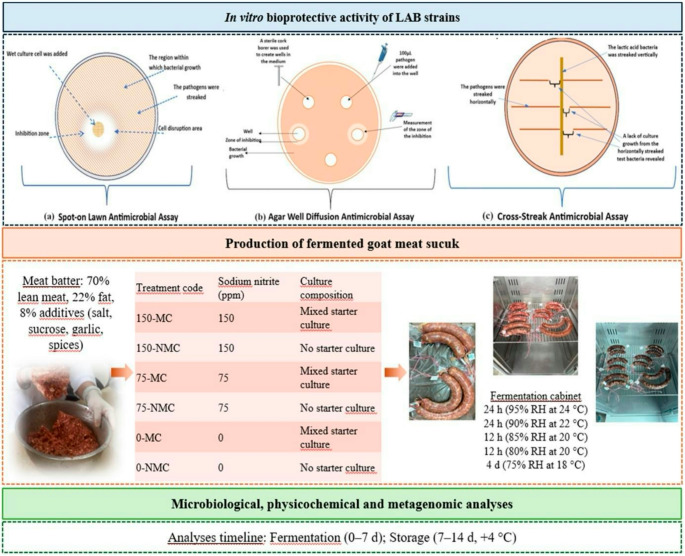
Schematic flowchart of fermented goat meat sucuk production, analyses, and *in vitro* assays

The in vitro bioprotective activity was evaluated using three different assay, as shown in Fig. 1. Each experiment was performed in triplicate. The cross-streak assay was performed by streaking active LAB cultures vertically and pathogen bacteria horizontally on the same agar plate (Postich and Kiser 2018). Strains were prepared and standardized as previously described. Inhibition was indicated by the absence of growth of horizontally streaked test bacteria where they intersected with the LAB streak at the end of the at 37˚C for 24 h incubation. In the spot-on lawn assay, freshly grown LAB cultures (20 µL) were spotted onto semi-solid agar (1% agar) inoculated with pathogen bacteria. Inhibition zones around the LAB spots were measured after incubation for 24 h at 37˚C for bacteria and 72 h at 30˚C for *C. albicans* (Büyüksırıt Bedir and Kuleaşan 2022). Agar well diffusion assay, the density of both groups was adjusted to 0.5 McFarland. The pathogen microorganisms were inoculated at 1% into cooled agar and poured into Petri dishes (~ 25 mL per 90 mm). After solidification, 9 mm wells were aseptically created using a sterile borer, and 100 µL of LAB culture was added to each well. Dimethyl sulfoxide (DMSO, 100%) was used as the negative control, and the presence of a clear zone surrounding the wells was considered indicative of bioprotective activity (Magaldi et al. 2004).

### Production of fermented goat meat sucuk

Goat hind leg meat was collected from female animals aged 1.5 to 2 years (18–20 kg live weight), slaughtered under commercial conditions. Carcasses were held for 24 h postmortem before sample collection. Meat from three hind legs was used, yielding approximately 1–1.5 kg of tissue each. Two batches of sucuk batter were prepared, weighing approximately 1.5 kg. The batter formulation comprised 70% lean meat and 22% beef fat, along with 2.5% NaCl, 0.5% sucrose, 1.5% garlic powder, and 3.5% spice mix (Bilecen Şen and Ertürkmen 2025). The sucuk batter was divided into six portions of 250 g each and formulated according to treatment specifications. The sucuk formulations then varied according to the presence or absence of these MC (inoculated at 1.5 × 10^8^ CFU/g) and sodium nitrite addition at 0, 75, and 150 ppm, resulting in distinct treatments combining mixed cultures and different nitrite levels (Fig. 1). The sucuk dough was rested at + 4˚C for approximately 6 h. Then, semi-permeable collagen casings (40 mm) were soaked in water at 15–20˚C for 5–10 min and carefully filled to prevent any air gaps (approximately two sucuk of 125 g each per treatment). After filling, the sucuk samples underwent a balancing phase, resting at room temperature for 2–3 h. Fermentation was carried out in an automatically controlled fermentation cabinet (Nüve GC 401, Turkey) with predefined temperature and relative humidity set points, using a stepwise fermentation program under the following conditions: 24 h at 24˚C with 95% humidity, 24 h at 22˚C with 90% humidity, 12 h at 20˚C with 85% humidity, 12 h at 20˚C with 80% humidity, and finally 4 days at 18˚C with 75% humidity. The stepwise fermentation program was established based on preliminary trials, and fermentation completion was defined by the completion of this predefined program. After fermentation, samples were stored under aerobic refrigerated conditions at + 4˚C for 14 days. All analyses were performed during fermentation (days 0 and 7) and storage (days 7 and 14).

### Culturable viable microorganism composition

All sucuk samples (10 g) were homogenized in 90 mL of sterile peptone water using a laboratory stomacher blender (Interscience BagMixer 400, France). Serial decimal dilutions were prepared aseptically, and the spread plate method was applied to enumerate culturable viable microorganisms. Selective agar media were used to quantify microbial groups as follows: Total mesophilic aerobic bacteria (TMAB) were enumerated on Plate Count Agar (PCA; Merck, Germany) after incubation at 30˚C for 48 h. LAB were enumerated using modified MRS Agar supplemented with CuSO₄ (6 µg/mL) and incubated at 30˚C for 48 h (Thompson et al. ro^2025^). Mold-yeast counts were enumerated on Rose Bengal Chloramphenicol Agar (RBCA), incubated at 25˚C for 3–5 d. Presumptive *Enterobacteriaceae* were detected on Violet Red Bile Agar (VRB; Merck, Germany), with plates incubated at 37˚C for 24 h; dark red to purple colonies surrounded by precipitation zones were counted (Barer and Harwood 1999).

### Physicochemical analyses

Physicochemical analyses of goat meat sucuk were performed on selected fermentation and storage days in two independent replicates, with duplicate measurements taken within each replicate. pH was measured using a digital pH meter (model HI 99163, Hanna Instruments, Romania), calibrated with standard pH 4 and pH 7 buffer solutions. Water activity (a_w_) values were determined using a water activity meter (Novasina AG Lab Swift, Switzerland). Total color differences (ΔE^*^) were calculated from CIE L^*^, a^*^, and b^*^ values, which were measured immediately after removing the sucuk samples from their casings to capture their color prior to any additional blooming. Color evaluation was conducted using a colorimeter (Konica-Minolta CR400, Osaka, Japan) equipped with a D65 light source, an 8.0 mm aperture, and a 10˚ standard observer angle.

### Metagenomic analysis

Metagenomic profiling of the 75-MC group was performed by targeting the bacterial 16 S rRNA gene (V3–V4 region) and the fungal ITS2 region at BM Labosis Laboratory (Ankara, Turkey). Amplicon library preparation and paired-end sequencing (2 × 250 bp) were conducted on the Illumina MiSeq platform. Bioinformatic analyses were carried out using QIIME2 (q2cli v2024.10.1) (Bolyen et al. 2019). Sequence quality was assessed, demultiplexed based on barcode information, and primer-trimmed prior to downstream analyses. Denoising, chimera removal, and amplicon sequence variant (ASV) inference were performed using the DADA2 plugin with parameters optimized for Illumina paired-end amplicon data (Callahan et al. 2016). Samples were rarefied to a uniform sequencing depth to minimize bias associated with unequal read counts. Taxonomic classification was conducted using the QIIME2 feature-classifier plugin against the SILVA reference database (v138) for bacterial communities and the UNITE database for fungal ITS sequences, applying a confidence threshold of 0.7. Host-derived sequences were excluded during taxonomic filtering. Taxonomic composition was visualized using Krona (q2-krona v1.0.2) (Ondov et al. 2011).

### Statistical analysis

The experiment was conducted using two independent biological replicates (separate production batches), each including six different goat meat sucuk treatments. For each sucuk sample and sampling day, analytical determinations were performed in duplicate (technical parallels). For statistical analysis, the mean of the technical replicates was calculated and used as the observational unit, resulting in four observations (*n* = 4) per treatment × day combination. Statistical analysis was performed using the General Linear Model (GLM) procedure in MINITAB software (version 16.0, Minitab Inc., USA). Two-way ANOVA was applied to evaluate the effects of treatment and day, as well as their interaction, on physicochemical and microbiological parameters. When significant effects were detected, Tukey’s post hoc test was used for multiple comparisons (*P* < 0.05). For in vitro analyses, one-way ANOVA followed by Tukey’s test was applied at the same significance level (*P* < 0.05). Data are expressed as mean values ± standard error (SE).

## Result and discussion

### *In vitro* bioprotective activity of LAB strains

**Fig. 2:**
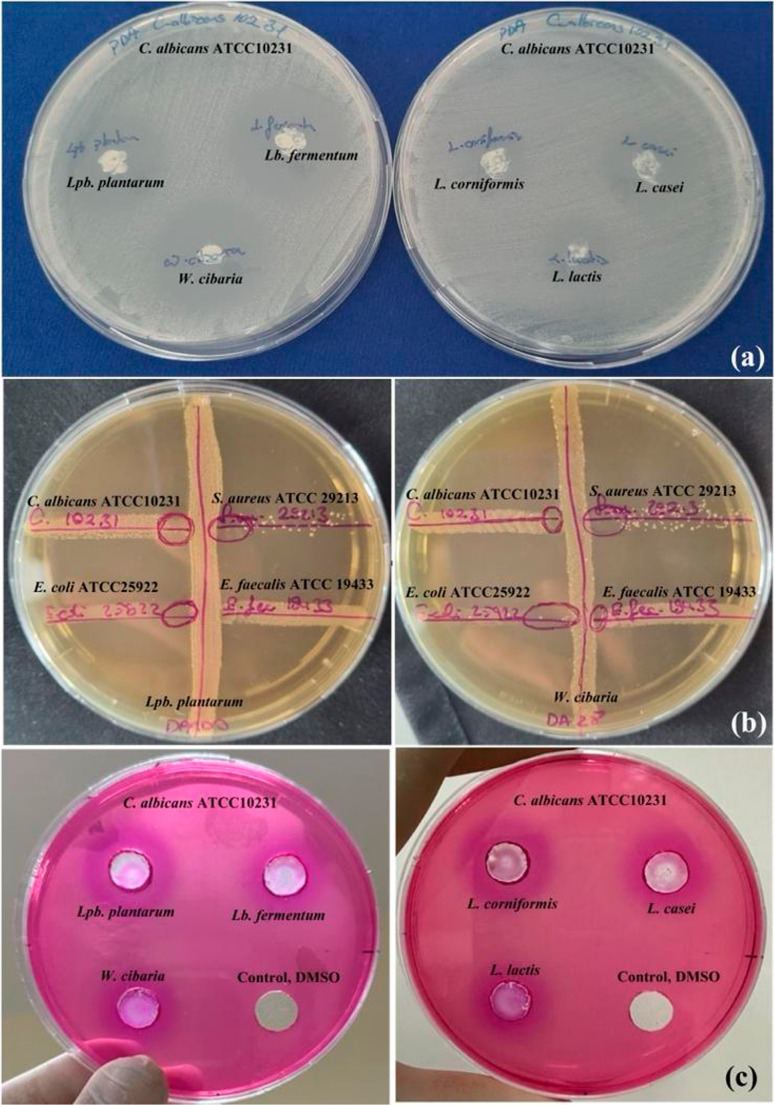
Photographs of i*n vitro* bioprotective activity results from different assays: (**a**) spot-on lawn- LAB results against *C. albicans* (ATCC10231), (**b**) cross-streak- *Lpb plantarum* and *W. cibaria* results against *C. albicans* (ATCC10231), *S. aureus* (ATCC29213), *E. coli* (ATCC25922) and *E. faecalis* (ATCC 19433) and (**c**) agar well diffusion- LAB results against *C. albicans* (ATCC10231)

Six LAB strains (*L. casei*, *L. coryniformis*, *Lb. fermentum*, *Lpb*. *plantarum*, *L. lactis*, and *W. cibaria*) were selected based on agar-based assays and previous evidence demonstrating the absence of antagonistic interactions, bacteriocin production (Alp 2018), or unexpected viability changes when used in combination (Alp and Kuleaşan 2020; Ertürkmen et al. 2024a, b). Their bioprotective activity against bacterial and yeast pathogens was evaluated using agar well diffusion, spot-on lawn, and cross-streak assays. Significant strain-dependent differences were observed within each assay (*P* < 0.05; Table 1; Fig. 2). Because these assays are based on different principles, inhibition zone diameters were evaluated within each method rather than compared across assays.

**Table 1:** Antimicrobial effects of lactic acid bacteria against various pathogens using different *in vitro* methods (zone diameters, mm)

Method	Lactic acid bacteria	Pathogen microorganism							
		*E. coli* (ATCC 25922)	*S. aureus* (ATCC 29213)	*S. aureus* (ATCC 6538)	*S.* Typhimurium (ATCC 14028)	*L. monocytogenes* (ATCC 7644)	*E. faecalis* (ATCC 19433)	*C. albicans* (ATCC 14053)	*C. albicans* (ATCC 10231)
Agar Well Diffusion	*L. casei*	10.5 ± 0.3^e^	9.5 ± 0.4^e^	11.3 ± 0.3^c^	9.5 ± 0.3^cd^	9.5 ± 0.5^ab^	11.3 ± 0.4^cd^	15.5 ± 0.0^c^	7.5 ± 0.3^d^
	*L. coryniformis*	10.0 ± 0.3^e^	10.5 ± 0.2^e^	8.5 ± 0.3^d^	8.0 ± 0.3^de^	7.5 ± 0.3^c^	12.5 ± 0.1^c^	12.5 ± 0.3^d^	9.5 ± 0.3^c^
	*Lb. fermentum*	27.0 ± 0.3^b^	18.5 ± 0.6^c^	20.5 ± 0.0^b^	11.5 ± 0.0^ab^	8.5 ± 0.3^bc^	17.5 ± 0.0^b^	24.0 ± 0.3^b^	31.0 ± 0.0^b^
	*Lpb. plantarum*	33.5 ± 0.2^a^	44.0 ± 0.0^a^	22.5 ± 0.6^a^	12.5 ± 0.6^a^	8.0 ± 0.2^bc^	18.5 ± 0.6^ab^	22.0 ± 0.4^b^	33.5 ± 0.6^a^
	*Leu. lactis*	13.5 ± 0.6^d^	13.0 ± 0.3^d^	7.5 ± 0.3^d^	7.5 ± 0.3^e^	10.5 ± 0.6^a^	10.5 ± 0.0^d^	11.0 ± 0.3^d^	8.0 ± 0.4^cd^
	*W. cibaria*	23.5 ± 0.3^c^	21.0 ± 0.3^b^	20.0 ± 0.6^b^	10.5 ± 0.3^bc^	9.5 ± 0.3^ab^	19.5 ± 0.0^a^	28.5 ± 0.4^a^	32.0 ± 0.0^ab^
Cross-Streak	*L. casei*	5.5 ± 0.1^bc^	4.5 ± 0.2^b^	3.5 ± 0.1^b^	2.5 ± 0.0^a^	2.5 ± 0.0^a^	3.5 ± 0.0^ab^	3.5 ± 0.0^ab^	2.5 ± 0.3^a^
	*L. coryniformis*	4.5 ± 0.2^c^	3.5 ± 0.3^b^	4.5 ± 0.2^b^	2.5 ± 0.1^a^	4.0 ± 0.3^a^	3.5 ± 0.1^ab^	3.5 ± 0.3^ab^	3.5 ± 0.4^a^
	*Lb. fermentum*	8.5 ± 0.3^b^	4.0 ± 0.2^b^	4.0 ± 0.3^b^	3.5 ± 0.3^a^	2.5 ± 0.2^a^	3.0 ± 0.3^ab^	2.0 ± 0.5^b^	3.0 ± 0.0^a^
	*Lpb. plantarum*	5.5 ± 0.3^bc^	5.0 ± 0.0^b^	4.5 ± 0.3^b^	4.5 ± 0.2^a^	3.0 ± 0.2^a^	1.5 ± 0.3^b^	5.0 ± 0.3^a^	1.5 ± 0.2^a^
	*Leu. lactis*	5.0 ± 0.1^c^	2.5 ± 0.3^b^	5.0 ± 0.3^b^	3.0 ± 0.1^a^	3.5 ± 0.4^a^	5.5 ± 0.5^a^	2.5 ± 0.3^ab^	2.5 ± 0.3^a^
	*W. cibaria*	14.5 ± 0.1^a^	9.5 ± 0.1^a^	8.0 ± 0.6^a^	3.5 ± 0.0^a^	5.0 ± 0.1^a^	3.5 ± 0.0^ab^	3.0 ± 0.1^ab^	2.0 ± 0.0^a^
Spot-on Lawn	*L. casei*	10.5 ± 0.3^d^	8.5 ± 0.2^e^	11.3 ± 0.2^c^	10.5 ± 0.6^cd^	8.5 ± 0.5^c^	11.8 ± 0.0^b^	27.0 ± 0.4^b^	18.5 ± 0.4^d^
	*L. coryniformis*	9.0 ± 0.6^d^	12.0 ± 0.2^d^	10.0 ± 0.1^c^	11.0 ± 0.3^c^	9.5 ± 0.3^c^	13.0 ± 0.1^b^	16.0 ± 0.5^c^	12.5 ± 0.3^e^
	*Lb. fermentum*	27.5 ± 0.0^b^	30.5 ± 0.2^b^	27.0 ± 0.4^b^	11.5 ± 0.2^bc^	18.5 ± 0.1^a^	25.5 ± 0.3^a^	30.5 ± 0.1^a^	43.0 ± 0.2^a^
	*Lpb. plantarum*	33.5 ± 0.1^a^	44.5 ± 0.3^a^	34.5 ± 0.0^a^	17.5 ± 0.5^a^	17.0 ± 0.2^ab^	22.0 ± 0.2^a^	24.0 ± 0.2^b^	33.5 ± 0.1^c^
	*Leu. lactis*	14.5 ± 0.1^c^	15.5 ± 0.2^c^	5.5 ± 0.0^d^	7.5 ± 0.3^d^	12.0 ± 0.2^c^	10.0 ± 0.0^b^	18.0 ± 0.5^c^	14.5 ± 0.3^e^
	*W. cibaria*	27.5 ± 0.4^b^	28.5 ± 0.3^b^	25.5 ± 0.5^b^	14.5 ± 0.0^ab^	15.5 ± 0.1^b^	24.5 ± 0.3^a^	32.0 ± 0.1^a^	37.5 ± 0.2^b^

a-e – Means with different letters in a column differ statistically significantly at *P*-value; ^*^P < 0.05. The results are presented as mean ± standard error. Statistical comparisons were performed only among LAB strains within the same assay and against the same pathogen

In the agar well diffusion assay, *Lpb. plantarum* exhibited the largest inhibition zones against most tested pathogens, including *E. coli*,* S. aureus* (ATCC 29213 and ATCC 6538), *S. Typhimurium*, and *C. albicans* (ATCC 10231). In the same assay, *W. cibaria* and *Lb. fermentum* showed significant anti-yeast activity against *C. albicans* (ATCC 10231) (32.0 ± 0.0 mm and 31.0 ± 0.0 mm, respectively), while *W. cibaria* exhibited the highest inhibitory effect against *E. faecalis* (19.5 ± 0.0 mm) (*P* < 0.05). Similarly, in the spot-on lawn assay, *Lpb. plantarum* showed strong inhibitory activity against *E. coli* and *S. aureus*. Inhibition zones against *S.* Typhimurium and *E. faecalis* ranged from 17.5 to 44.5 mm (*P* < 0.05). Furthermore, *Lb. fermentum* and *W. cibaria* demonstrated significant anti-yeast activity against *C. albicans* (ATCC 10231) (43.0 ± 0.2 mm and 37.5 ± 0.2 mm, respectively), whereas *L. casei*, *L. coryniformis* and *L. lactis* consistently produced smaller inhibition zones (*P* < 0.05). In the cross-streak assay, *W. cibaria* displayed the strongest inhibitory activity against *E. coli* (14.5 ± 0.1 mm) and *S. aureus* (ATCC 29213 and ATCC 6538; 9.5 ± 0.1 mm and 8.0 ± 0.6 mm, respectively), followed by *Lb. fermentum* against *E. coli* (8.5 ± 0.3 mm) (*P* < 0.05).

Taken together, although absolute inhibition zone sizes obtained from different assay formats are not directly comparable, the consistent and reproducible inhibitory performance of *Lpb. plantarum*, *Lb. fermentum*, and *W. cibaria* across all assays supported their selection as the mixed culture (MC) for application in fermented sucuk production. Several studies have previously reported the bioprotective potential of LAB strains against foodborne pathogens and spoilage microorganisms. For instance, *Lb. fermentum* has been shown to inhibit *Geotrichum candidum*, producing inhibition zones of 16.60 ± 0.70 mm (Behbahani et al. 2023). In addition, the RSB9 strain suppressed *S. aureus* and *L. monocytogenes* (Alizadeh-Behbahani et al. 2024), while the LAB-1 strain was effective against *E. coli* and *Bacillus cereus* (Tarannum et al. 2024). Consistent with these findings, *Lpb. plantarum*, *Lb. fermentum*, and *W. cibaria* exhibited the strongest inhibitory effects among the six LAB strains tested, further supporting their selection as the mixed culture (MC) for use in fermented sucuk production.

### Changes in microorganism composition of fermented goat meat sucuk

The results of culturable viable microorganism counts in fermented goat meat sucuk are presented in Table 2. Day had a significant effect on all microbial counts during both fermentation and storage (*P* < 0.05). Treatment effects were mostly significant, except for TMAB during fermentation (*P* > 0.05). A significant treatment × day interaction was observed at the fermentation stage for most groups (*P* < 0.05), but not during storage (*P* > 0.05), indicating greater variability in microbial growth during fermentation.

**Table 2:** Effects of sodium nitrite level, culture composition, and fermentation/storage day on culturable viable microorganism counts (log CFU/g) in sucuk samples

Microorganisms	Treatment	Fermentation (day)		Mean ± SE	Storage (day)		Mean ± SE
		0	7		7	14	
Total mesophilic aerobic bacteria (TMAB)	150-MC	7.32^bc^	8.69^a^	8.01 ± 0.29	8.51	8.45	8.48 ± 0.06^A^
	150-NMC	7.31^bc^	7.91^abc^	7.61 ± 0.29	7.35	7.79	7.57 ± 0.15^C^
	75-MC	7.22^c^	8.56^ab^	7.89 ± 0.34	8.37	8.69	8.53 ± 0.08^A^
	75-NMC	7.69^abc^	7.38^bc^	7.53 ± 0.13	7.36	7.86	7.82 ± 0.34^BC^
	0-MC	7.21^c^	8.23^abc^	7.72 ± 0.21	8.26	8.39	8.33 ± 0.04^AB^
	0-NMC	7.50^abc^	7.82^abc^	7.66 ± 0.15	7.45	7.44	7.45 ± 0.05^C^
	Mean ± SE	7.37 ± 0.13^B^	8.10 ± 0.11^A^		7.88 ± 0.14^B^	8.17 ± 0.10^A^	
*P*-value	T	0.474			< 0.05		
	D	< 0.05			< 0.05		
	T×D	< 0.05			0.202		
*Lactobacillaceae*	150-MC	7.36^cde^	8.76^a^	8.06 ± 0.28^A^	8.14^abc^	8.20^abc^	8.17 ± 0.07^AB^
	150-NMC	6.15^f^	7.75^bcd^	6.95 ± 0.32^B^	7.26^d^	7.11^d^	7.19 ± 0.03^D^
	75-MC	7.24^de^	8.51^ab^	7.88 ± 0.25^A^	8.41^ab^	8.57^a^	8.49 ± 0.04^A^
	75-NMC	6.69^ef^	7.08^de^	6.89 ± 0.22^B^	7.30^d^	7.93^bc^	7.61 ± 0.18^C^
	0-MC	7.04^de^	8.18^abc^	7.61 ± 0.24^A^	7.86^c^	8.21^abc^	8.03 ± 0.09^B^
	0-NMC	6.80^ef^	7.08^de^	6.94 ± 0.08^B^	7.94^bc^	7.86^c^	7.90 ± 0.03^BC^
	Mean ± SE	6.88 ± 0.11^B^	7.89 ± 0.14^A^		7.82 ± 0.09^B^	7.98 ± 0.10^A^	
*P*-value	T	< 0.05			< 0.05		
	D	< 0.05			< 0.05		
	T×D	< 0.05			< 0.05		
Mold and yeast	150-MC	4.74^cde^	4.61^e^	4.68 ± 0.13^C^	4.68	4.63	4.65 ± 0.08^B^
	150-NMC	4.71^de^	5.30^ab^	5.00 ± 0.12^BC^	5.66	5.68	5.67 ± 0.08^A^
	75-MC	4.72^de^	5.27^abc^	5.00 ± 0.11^BC^	5.28	5.37	5.33 ± 0.09^A^
	75-NMC	5.21^abcd^	5.31^ab^	5.26 ± 0.08^AB^	5.30	5.70	5.50 ± 0.07^A^
	0-MC	4.99^bcde^	5.53^a^	5.26 ± 0.12^AB^	5.38	5.55	5.47 ± 0.04^A^
	0-NMC	5.17^abcd^	5.58^a^	5.37 ± 0.09^A^	5.38	5.76	5.57 ± 0.13^A^
	Mean ± SE	4.92 ± 0.07^B^	5.27 ± 0.07^A^		5.28 ± 0.07^B^	5.45 ± 0.09^A^	
*P*-value	T	< 0.05			< 0.05		
	D	< 0.05			< 0.05		
	T×D	< 0.05			0.252		
*Enterobacteriaceae*	150-MC	3.17^cd^	2.25^e^	2.71 ± 0.19^D^	2.20^bc^	1.50^e^	1.85 ± 0.14^C^
	150-NMC	3.62^ab^	2.46^e^	3.04 ± 0.22^C^	2.27^bc^	2.11^bc^	2.19 ± 0.09^B^
	75-MC	3.46^abc^	2.33^e^	2.89 ± 0.22^CD^	1.97^cd^	1.59^de^	1.78 ± 0.07^C^
	75-NMC	3.74^a^	2.53^e^	3.13 ± 0.23^BC^	2.30^bc^	2.22^bc^	2.25 ± 0.04^B^
	0-MC	3.77^a^	2.96^d^	3.37 ± 0.15^AB^	2.48^b^	2.33^bc^	2.41 ± 0.06^B^
	0-NMC	3.78^a^	3.34^bcd^	3.56 ± 0.11^A^	3.27^a^	3.48^a^	3.37 ± 0.08^A^
	Mean ± SE	3.59 ± 0.05^A^	2.64 ± 0.09^B^		2.42 ± 0.09^A^	2.20 ± 0.14^B^	
*P*-value	T	< 0.05			< 0.05		
	D	< 0.05			< 0.05		
	T×D	< 0.05			< 0.05		

a-f – Means with different letters differ statistically significantly at *P*-value (significance of T×D interaction); A-D – Means with different letters in a row or column differ statistically significantly at *P*-value (significance of main factors - T or D). SE – standard error; T – treatment; D – day; T×D – treatment×day interaction

TMAB counts ranged from 7.21 to 8.69 log CFU/g during fermentation and from 7.36 to 8.69 log CFU/g during storage. All treatments exhibited an increase in TMAB during the fermentation period, particularly those with MC (*P* < 0.05). This increase likely reflects the growth of LAB within the total mesophilic aerobic flora (Yalçın and Ertürkmen 2024). In contrast, non-MC groups showed lower TMAB levels at the end of fermentation (*P* > 0.05). During storage, TMAB levels generally increased, especially in groups with added MC. Starter culture-added groups, such as 150-MC and 75-MC, maintained the highest TMAB counts (8.48 and 8.53 log CFU/g), while the lowest count was recorded in 0-NMC (7.45 log CFU/g) (*P* < 0.05). These results are consistent with previous studies reporting TMAB counts exceeding 8.0 log CFU/g during the advanced stages of ripening in dry-fermented sausages (Soyer et al. 2005; Yalçın and Ertürkmen 2024).

During fermentation, *Lactobacillaceae* counts ranged from 6.15 to 8.76 log CFU/g, while during storage ranged between 7.11 and 8.57 log CFU/g. Treatments added with MC showed significantly higher LAB levels than non-MC groups (*P* < 0.05). By the end of fermentation, 150-MC, 75-MC, and 0-MC all exceeded 8.0 log CFU/g, demonstrating strong LAB growth. These results indicated that reducing nitrite from 150 to 75 ppm did not affect LAB proliferation when the MC was included. In contrast, the non-MC treatments (150-NMC, 75-NMC, and 0-NMC) had lower LAB levels, emphasizing the critical role of starter cultures for effective fermentation. Similar observations have been reported previously, where starter cultures supported LAB growth and acidification under nitrite-reduced conditions. (Laranjo et al. 2019; Bozkurt and Erkmen 2002), The strong performance of the MC can be explained by the aciduric and metabolically adaptable properties of the selected strains, which facilitate rapid carbohydrate metabolism and competitive advantage under low-nitrite conditions (Fusco et al. 2015). Consistent with this, MC groups also exhibited the lowest pH values (< 4.80), confirming their acidogenic capacity (Table 3). LAB counts remained above 8.0 log CFU/g even after storage, showing that the bioprotective effect persisted during refrigeration. Comparable microbial patterns in dry-fermented sucuk have also been described by Pavli et al. (2020). The combination of *Lb. fermentum* and *W. cibaria* with *Lpb. plantarum* may have contributed to early microbial establishment and accelerated acidification across nitrite levels. This effect may be related to strain-specific metabolic activities or competitive dominance of certain LAB during fermentation, as previously reported in mixed-culture fermentations (Leroy and De Vuyst 2004; Laranjo et al. 2019).

**Table 3:** Variation in physicochemical properties of sucuk samples depending on sodium nitrite level, culture composition, and fermentation/storage day

Parameters	Treatment	Fermentation (day)		Mean ± SE	Storage (day)		Mean ± SE
		0	7		7	14	
pH	150-MC	5.38^a^	4.68^g^	5.03 ± 0.13^BC^	4.70^d^	4.72^d^	4.71 ± 0.01^D^
	150-NMC	5.25^ab^	4.96^def^	5.11 ± 0.06^B^	4.87^c^	4.95^b^	4.91 ± 0.02^C^
	75-MC	5.05^cde^	4.62^g^	4.84 ± 0.08^D^	4.67^d^	4.74^d^	4.71 ± 0.02^D^
	75-NMC	5.15^bc^	4.90^ef^	5.02 ± 0.05^BC^	4.99^b^	4.97^b^	4.98 ± 0.02^B^
	0-MC	5.13^bc^	4.86^f^	4.99 ± 0.05^C^	4.92^bc^	4.96^b^	4.94 ± 0.01^BC^
	0-NMC	5.37^a^	5.11^bcd^	5.24 ± 0.05^A^	5.09^a^	5.17^a^	5.13 ± 0.00^A^
	Mean ± SE	5.22 ± 0.03^A^	4.85 ± 0.04^B^		4.87 ± 0.03^B^	4.92 ± 0.03^A^	
*P*-value	T	< 0.05			< 0.05		
	D	< 0.05			< 0.05		
	T×D	< 0.05			< 0.05		
Water activity (a_w_)	150-MC	0.934^a^	0.848^i^	0.891 ± 0.025^B^	0.834^d^	0.801^e^	0.817 ± 0.010^D^
	150-NMC	0.928^b^	0.854^h^	0.891 ± 0.021^B^	0.861^c^	0.861^c^	0.861 ± 0.000^C^
	75-MC	0.912^d^	0.867^g^	0.889 ± 0.013^B^	0.861^c^	0.860^c^	0.860 ± 0.008^C^
	75-NMC	0.926^bc^	0.871^f^	0.898 ± 0.016^A^	0.862^c^	0.860^c^	0.861 ± 0.002^C^
	0-MC	0.926^bc^	0.872^f^	0.899 ± 0.016^A^	0.871^a^	0.861^c^	0.866 ± 0.003^A^
	0-NMC	0.925^c^	0.875^e^	0.900 ± 0.014^A^	0.868^b^	0.860^c^	0.864 ± 0.002^B^
	Mean ± SE	0.925 ± 0.001^A^	0.864 ± 0.002^B^		0.859 ± 0.003^A^	0.850 ± 0.005^B^	
*P*-value	T	< 0.05			< 0.05		
	D	< 0.05			< 0.05		
	T×D	< 0.05			< 0.05		
Total color differences (ΔE^*^)	150-MC	10.43 ± 1.05			6.15 ± 1.49^d^		
	150-NMC	11.29 ± 1.48			6.75 ± 0.81^d^		
	75-MC	10.33 ± 1.62			7.38 ± 1.31^c^		
	75-NMC	11.79 ± 1.01			8.09 ± 1.60^b^		
	0-MC	11.13 ± 1.42			9.56 ± 1.45^a^		
	0-NMC	11.95 ± 1.75			9.75 ± 1.82^a^		
*P*-value		0.966			< 0.05		

a-i – Means with different letters differ statistically significantly at *P*-value (significance of T×D interaction); A-D – Means with different letters in a row or column differ statistically significantly at *P*-value (significance of main factors - T or D). SE – standard error; T – treatment; D – day; T×D – treatment×day interaction

Mold-yeast counts increased significantly in all treatments during fermentation (*P* < 0.05). At the end of this period, only the 150-MC showed a lower count (*P* < 0.05), while the other ranged between 5.27 and 5.58 log CFU/g. This reduction seems to be related to the combined effect of the starter culture and the high nitrite level (Bilecen Şen and Ertürkmen 2025). During storage, 150-MC maintained the lowest mold-yeast counts (*P* < 0.05). In contrast, the remaining treatments exceeded 5 log CFU/g, with the highest counts detected in 0-NMC. Comparable results were noted by Yilmaz-Topcam et al. (2024), who found mold-yeast counts around 4–5 log CFU/g in traditionally fermented sausages inoculated with starter cultures. These findings suggest a possible synergistic effect of nitrite and the MC in limiting fungal growth. This effect cannot be attributed to low pH alone, as many yeast species are acid-tolerant, but may instead be associated with the combined action of nitrite, organic acids, and other inhibitory metabolites produced by LAB, potentially creating unfavorable conditions for fungal growth (Leroy and De Vuyst 2004), particularly in treatments such as 150-MC with lower final pH values (< 4.80; Table 3).

*Enterobacteriaceae* counts decreased significantly during fermentation in all treatments (*P* < 0.05), which may reflect the development of unfavorable conditions for Gram-negative bacteria during the fermentation process. At the end of fermentation, counts ranged from 2.25 to 3.34 log CFU/g, with the lowest levels observed in treatments containing 150 or 75 ppm nitrite, regardless of MC addition. This decline appears to be linked to the bioprotective effect of nitrite, which enhances microbial competition and promotes acidification, creating unfavorable conditions for *Enterobacteriaceae*. By the end of storage, *Enterobacteriaceae* were undetectable (< 2 log CFU/g) in 150-MC and 75-MC, showing that the hurdle strategy remained effective during refrigeration. In contrast, the highest count was found in 0-NMC, which lacked both nitrite and MC (*P* < 0.05). This suggests that the absence of these hurdles allowed the survival or re-growth of spoilage-associated Gram-negative bacteria during storage. Even in 0-MC, *Enterobacteriaceae* persisted at 2.33 log CFU/g, underlining the importance of nitrite for microbial control. This finding highlights the key role of nitrite in strengthening microbial count. Our results agree with earlier reports showing that LAB strains such as *W. cibaria* and *Lb. fermentum* can inhibit pathogens and spoilage microorganisms through organic acids, hydrogen peroxide, and bacteriocin-like compounds (Fusco et al. 2015; Fessard and Remize 2017). The reductions may also be related to lower pH and decreased water activity in MC groups (Table 3), which provide an additional antimicrobial barrier (Wang et al. 2021; Akköse et al. 2023). These findings highlight the importance of combining nitrite with the MC to maintain microbial safety and stability in fermented goat meat sucuk.

### Physicochemical changes in fermented goat meat sucuk

Table 3 shows the pH, water activity (a_w_), and total color difference (ΔE^*^) values of the sucuk samples. Significant effects of day and treatment were observed for pH and a_w_ during fermentation and storage periods (*P* < 0.05), and a treatment × day interaction was also detected (*P* < 0.05). For ΔE^*^ values, no treatment effects was found during fermentation (*P* > 0.05). However, significant differences were detected during storage (*P* < 0.05), indicating that color change was influenced more by storage than by the fermentation stage.

The initial pH on day 0 ranged from 5.05 to 5.38 across treatments. By the end of fermentation, pH had reduced significantly in all groups (*P* < 0.05), with the lowest values in MC-added treatments such as 150-MC and 75-MC. These results are consistent with the high LAB counts reported in Table 2 and reflect the acidogenic activity of the starter culture, which accelerates pH decline in fermented meats (Mani-López et al. 2024; Carneiro et al. 2024). In contrast, the 0-NMC, lacking both nitrite and mixed culture, maintained the highest pH at the end of fermentation, indicating reduced acidification capacity. During storage, MC-added groups sustained significantly lower pH values (< 4.80) compared to non-starter groups (*P* < 0.05). The lowest values during storage were again observed in 150-MC and 75-MC. This persistent low pH likely enhances product safety by creating conditions unfavorable to spoilage and pathogen bacteria (Toldrá and Hui 2014; Leroy et al. 2023). Furthermore, the combination of nitrite and mixed culture, as in 150-MC and 75-MC, has been reported to exert a synergistic effect in establishing a stable acidic environment, which suppresses the growth of undesirable microorganisms (Hwang et al. 2023). This is consistent with the reduced *Enterobacteriaceae* counts observed in these treatments (Table 2), highlighting the role of LAB-driven acidification in improving the microbial stability of sucuk.

Water activity significantly decreased during fermentation in all treatments (*P* < 0.05), reflecting moisture loss driven by fermentation and drying. Mixed culture addition did not markedly change a_w_ reduction trends during fermentation, but nitrite level and culture absence influenced the final a_w_ values, with the lowest observed in 150-MC (*P* < 0.05). Notably, 75-MC maintained similar a_w_ values at the end of fermentation and after storage, suggesting that reducing nitrite from 150 to 75 ppm did not compromise drying efficiency when MC was used. In contrast, the highest a_w_ occurred in 0-NMC, indicating that nitrite and mixed culture may enhance drying and moisture-binding capacity (Hwang et al. 2023). During storage, a_w_ declined in all treatments (*P* < 0.05). The lowest values were found in 50-MC, while 0-MC showed the highest. Lower a_w_ in nitrite-containing groups, especially those with starter cultures, can be explained by protein denaturation and to salt-driven water binding during fermentation. Starter cultures may also accelerate drying by lowering pH, which promotes further moisture loss (Sallan et al. 2020). In addition, a_w_ values below 0.86 are generally regarded as critical for microbiological stability in fermented sausages, explaining the longer shelf life observed in these groups (Kang and Yim 2025). Our findings are consistent with Mataragas et al. (2015), who showed that water reduction (a_w_) plays an important role in limiting pathogens such as *Salmonella* spp. in slowly acidified fermented sausages.

No significant differences in ΔE^*^ were detected among treatments during fermentation (*P* > 0.05), with values ranging from 10.33 to 11.95. This suggests that the main color development during fermentation was comparable across treatments, likely influenced by similar meat pigments and fermentation conditions (Han et al. 2024). However, during storage, ΔE^*^ values varied significantly (*P* < 0.05). The higher ΔE^*^ observed in nitrite-free groups highlights the essential role of sodium nitrite in stabilizing cured meat color through the formation of nitrosylated myoglobin pigments and the inhibition of lipid peroxidation (Sheng et al. 2025). MC did not show a direct impact on ΔE^*^, but its indirect effect via pH and oxidative stability may have contributed to lower color variation in certain treatments, consistent with prior reports. Specifically, studies have demonstrated that LAB starter cultures can protect proteins and lipids from oxidation, thereby enhancing color stability during storage (Palavecino Prpich et al. 2015; Stadnik et al. 2022).

### Taxonomic insights into bacterial diversity via 16 S rRNA (V3–V4) region

**Fig. 3:**
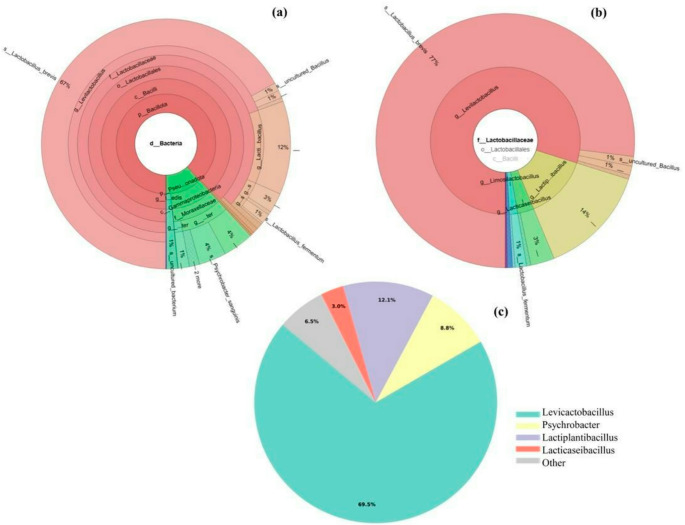
Krona visualization of bacterial diversities in nitrite-reduced fermented goat meat sucuk. Krona (**a**) highlights the dominance of lactic acid bacteria (LAB), while Krona (**b**) specifies the prevalence of the family *Lactobacillaceae*. Krona (**c**) illustrates the overall taxonomic group-level composition, confirming the LAB-driven microbiota structure and the hierarchical dominance of fermentative taxa over spoilage- and environment-associated microorganisms

Metagenomic sequencing of fermented goat meat sucuk generated 27.439 reads (41.7 Mbp) with lengths ranging from 1211 to 1642 bp and a mean GC content of 51%. After stringent quality filtering, 66 high-quality consensus sequences (mean 1473.7 bp) were retained, ensuring robust genus-level taxonomic resolution. Metagenomic profiling based on the 16 S rRNA (V3–V4) region revealed a diverse bacterial diversity predominantly resolved at the genus level. The microbiota was dominated by the phyla *Bacillota* and *Pseudomonadota*, with *Bacillota* accounting for 68–75% of the sequences across ripening stages, indicating the selective enrichment of LAB-associated taxa. ASVs affiliated with LAB–related genera were predominantly represented by *Levilactobacillus* (69.5%), followed by *Lactiplantibacillus* (12.1%), *Psychrobacter* (8.8%), and *Lacticaseibacillus* (3.0%). The Krona visualization (Fig. 3) illustrates the hierarchical dominance of LAB-associated genera and highlights the structured microbial diversity characterizing nitrite-reduced fermented goat meat sucuk.

Despite inoculation with a mixed culture comprising *Lactiplantibacillus*, *Limosilactobacillus*, and *Weissella*, the mature microbiota was predominantly dominated by *Levilactobacillus*, with *Lactobacillus brevis* emerging as the prevailing species. This compositional shift likely reflects ecological selection pressures associated with acidification dynamics, water activity, salt concentration, and the contribution of spice-derived indigenous LAB that are well adapted to the fermented meat matrix. Such observations are consistent with reports from fermented meat systems, particularly under nitrite-reduced conditions, where indigenous LAB exhibit strong adaptation to both raw materials and processing environments and may constrain starter culture proliferation through competitive exclusion and niche-based interactions (Carneiro et al. 2024). In agreement with previous studies, members of the genus *Weissella* appeared to function as early colonizers, potentially facilitating the initial stages of fermentation and community establishment (Kang et al. 2020; Lee et al. 2020; Liu et al. 2025). Importantly, the contribution of the MC should not be evaluated solely based on relative abundance, as non-proliferating or inactivated starter cells may still exert relevant technological and bioprotective functions through the production of metabolites, enzymes, bacteriocins or via structural components such as cell wall fragments, commonly referred to as postbiotics or paraprobiotics. Non-LAB genera such as *Staphylococcus*, *Brochothrix*, and *Clostridium* were detected at lower relative abundances. Members of the *Moraxellaceae* family were also observed, represented by the genus *Psychrobacter*, together with environmental and opportunistic genera including *Acinetobacter*, *Ignatzschineria*, and *Klebsiella*. Similarly, genera such as *Psychrobacter* and *Acinetobacter* have been identified across various meat processing environments and products, underscoring their environmental origin and potential relevance to product quality and safety, despite their low relative abundance in mature fermented meat microbiomes (Barcenilla et al. 2024).

### Characterization of fungal diversity via ITS2 gene region

**Fig. 4:**
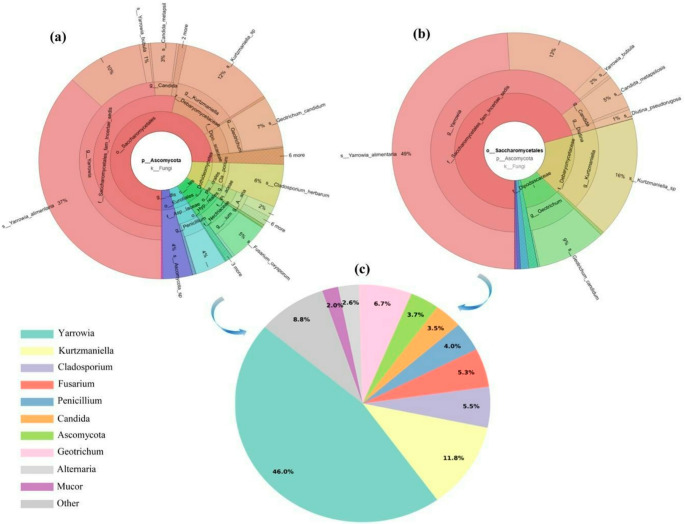
Krona visualization of fungal diversities in nitrite-reduced fermented goat meat sucuk. Krona (**a**) depicts the distribution of *Ascomycota* taxa, whereas Krona (**b**) shows the dominance of yeasts within the order *Saccharomycetales*, which accounted for more than 70% of the sequences. Krona (**c**) presents the taxonomic group-level composition, reflecting the structured fungal diversity of the product

Metagenomic profiling targeting the ITS2 region revealed a highly diverse fungal diversity in nitrite-reduced sucuk, with taxonomic assignments predominantly resolved at the genus level. Overall, the fungal ecosystem was largely dominated by yeasts belonging to the order *Saccharomycetales*, which accounted for over 70% of the total sequences detected. Within the phylum *Ascomycota*, the community structure was primarily characterized by the dominance of *Yarrowia* (46%) and *Kurtzmaniella* (11.8%), followed by *Geotrichum* (6.7%) and *Cladosporium* (5.5%). In addition, several genera typically associated with *Basidiomycota*, including *Penicillium*, *Candida*, *Alternaria*, and *Fusarium*, were identified at low relative abundances (< 1%). The relative distribution of *Ascomycota* and *Saccharomycetales* within the fungal microbiota is visually summarized in the Krona visualization (Fig. 4).

*Yarrowia* species have been widely reported in meat fermentation processes due to their pronounced lipolytic and proteolytic activities, which are closely linked to flavor development and increased aroma complexity (Flores et al. 2015; Mirbagheri et al. 2012). In this study, the marked dominance of *Yarrowia alimentaria*–related ASVs (49%) suggests that these yeasts may play a pivotal role in shaping the biochemical environment during the ripening of sucuk. The concurrent presence of *Kurtzmaniella* and *Geotrichum* further underscores the complexity of microbial interactions within the sucuk microbiota. Previous studies have associated *Kurtzmaniella* species with carbohydrate metabolism and ethanol production in fermented food matrices (Zeng et al. 2025), whereas *Geotrichum candidum* has been extensively linked to proteolytic and lipolytic activities contributing to texture and flavor development during ripening (Kamilari et al. 2023). Although *Basidiomycota*-associated yeasts were detected at relatively low abundances, their presence remains noteworthy given their reported bioprotective and technological functions in fermented food ecosystems. These low-abundance taxa likely reflect environmental inputs; nevertheless, they have been associated with oxidative stress tolerance and secondary metabolite production, suggesting potential supportive or stabilizing roles during sucuk ripening (Boekhout and Gueho 2002).

## Conclusion

The strategic application of a mixed culture composed of LAB affiliated with the genera *Lactiplantibacillus*, *Weissella*, and *Limosilactobacillus* under nitrite-reduced conditions was associated with a clear shift toward a LAB-dominated microbiota in fermented goat meat sucuk. Microbiota profiling demonstrated that the combination of 75 ppm nitrite with the mixed culture promoted bacterial diversities predominantly composed of LAB-related genera, particularly *Levilactobacillus*, *Lactiplantibacillus*, and *Lacticaseibacillus*, which are widely recognized as key functional members of fermented meat ecosystems. The fungal diversity during ripening was mainly represented by yeasts affiliated with the genera *Yarrowia* and *Kurtzmaniella*, highlighting their potential contribution to biochemical transformations associated with flavor and aroma development. Members of the genus *Weissella* were primarily associated with the early stages of fermentation, supporting their role as early colonizers in the establishment of the microbial community. Overall, these findings indicate that microbiota-targeted fermentation strategies, when combined with controlled nitrite reduction, can support microbial stability and technological performance in fermented meat products, offering a promising and scientifically grounded clean-label approach for the development of nitrite-reduced fermented meat formulations.

## Data Availability

The datasets generated during this study are partially presented in the figures and tables of this article. Additional metagenomic data that support the findings of this study are available from the corresponding author on reasonable request.
